# Integrative Analysis and Experimental Validation Identify Potential m6A-Related Biomarkers for Osteoporosis

**DOI:** 10.3390/genes17040458

**Published:** 2026-04-14

**Authors:** Zhenyang Wang, Yongqin Chen, Yuxuan Yang, Biteng Xu, Xiejia Jiao, Lei Qi

**Affiliations:** 1 Department of Orthopaedics, Qilu Hospital of Shandong University, Jinan 250012, China; a704615998@163.com (Z.W.); cyq1767698369@163.com (Y.C.); 15665705136@163.com (B.X.); 2 Department of Orthopaedics, The Second Qilu Hospital of Shandong University, Jinan 250012, China; 3 Traditional Chinese Medicine Hospital Affiliated to Xinjiang Medical University, Urumqi 830000, China

**Keywords:** osteoporosis, m6A regulators, immune microenvironment, RNA sequencing, Western blot

## Abstract

Background: This study investigates the role of N6-methyladenosine (m6A) regulators in osteoporosis (OP) and their interplay with the immune microenvironment, aiming to identify potential m6A-related biomarkers for OP risk assessment and treatment. Methods: Transcriptomic data from GEO datasets were analyzed for differential expression of 22 m6A regulators and immune infiltration patterns. Consensus clustering and m6Ascore grouping defined molecular subtypes, while machine learning algorithms identified potential biomarkers, leading to the construction and validation of a nomogram. Experimental validation involved peripheral blood monocytes (PBMCs) transcriptome sequencing and Western blot of bone tissue. Results: *FTO*, *HNRNPC*, and *METTL4* were upregulated, while *CBLL1* and *YTHDF2* were downregulated in OP, with two distinct m6A modification patterns and immune phenotypes identified. *METTL4*, *HIRA*, *MATN4*, and *YTHDF2* were selected as potential biomarkers, and the nomogram demonstrated favorable predictive performance in training and external datasets. Single-cell RNA sequencing confirmed the cellular distribution of these biomarkers. *HIRA* heterogeneity in Marrow Mesenchymal Stem Cells (BMSCs) was associated with distinct cell–cell communication patterns. Transcriptome sequencing confirmed *HIRA* RNA downregulation in OP PBMCs, and Western blot verified decreased HIRA protein in OP bone tissue. Conclusions: This study establishes a potential m6A-related biomarker signature for OP and provides multi-level experimental evidence that *HIRA* is a consistently downregulated biomarker, linking epigenetic modification to immune dysregulation in osteoporosis.

## 1. Introduction

Osteoporosis (OP) constitutes a chronic skeletal disorder severely impacting elderly populations [1], distinguished by diminished bone mass and deteriorated bone microarchitecture [2]. This disease clinically presents as heightened bone fragility with elevated fracture susceptibility, occurring even under minimal stress or spontaneously [3]. Postmenopausal and senior females exhibit significantly higher OP prevalence [4]. Systematic reviews have demonstrated that while osteoporosis is a global concern, its prevalence is disproportionately higher in postmenopausal women compared to the general population, whereas the rate in men is notably lower [5]. Currently recognized as a primary contributor to disease burden and fatal outcomes among the elderly, OP imposes substantial public health challenges and severe socioeconomic costs [6]. Implementing early risk assessment protocols and enhanced therapeutic strategies proves critical for delaying disease progression and mitigating OP-related complications [7].

Since the 1960s, the role of mRNA modification in various biological progress has been extensively studied [8]. More than 100 different forms of RNA modifications have been found [9]. It mainly includes N6-methyladenosine (m6A), 5-methylcytosine (m5C), 7-methylguanylate (m7G), N1-methyladenosine (m1A), and pseudouridine. m6A is a widely distributed modification regulated by a number of important proteins: writers, readers, and erasers [10]. Writers, also called methyltransferases, promote methylation of mRNA, and *METTL3*, *METTL14*, *WTAP* and *RBM15* are the main writers [11]. Readers, such as *IGF2BP2*, *IGF2BP3*, *YTHDC1*, *YTHDC2*, *YTHDF1*, *YTHDF2*, *YTHDF3*, and *FMR1*, are proteins that bind to mRNA to start particular biological process. Erasers, also known as demethylases, including *FTO*, erase m6A modification and regulate mRNAs metabolizations [10]. Various evidence demonstrated that m6A regulators are critical for RNA metabolism and involved in the physiological and pathological processes, including bone metabolism [12]. Sun Z et al. found that *METTL14* is positively related to bone formation in elderly women with serious osteoporotic fracture [13]. Wang J has reported that *FTO*-mediated m6A demethylation of *RUNX2* inhibits osteogenic potential of Bone Marrow Mesenchymal Stem Cells (BMSCs), and promotes osteoporosis [14]. The study also demonstrates that *METTL3*-mediated m6A methylation of *LINC00657* promotes the osteogenic potential of MSCs via miR-144-3p/BMPR1B axis [15].

Recently more and more evidence has demonstrated the potential role of m6A modification in the progression of OP [16,17,18]. Consequently, new reliable biomarkers and targeted therapies based on m6A hold considerable promise for clinical practice [19,20]. This study’s goal is to find out more about the role of m6A regulators and the molecular patterns they are linked to in OP so that we can see if they can be used to help with detection and treatment, which would lead to better ways of treating OP.

## 2. Results

**Figure 1:**
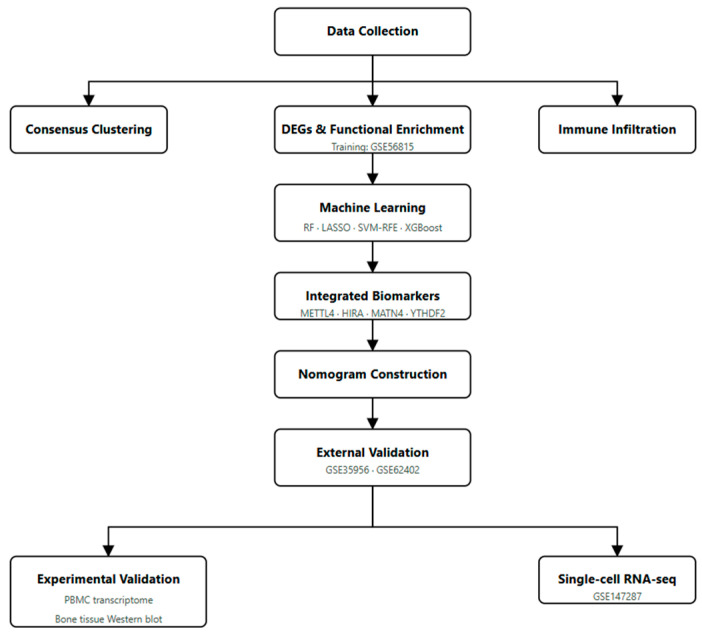
The overall analytical workflow.

The overall analytical workflow is summarized in Figure 1.

### 2.1. Identification of m6A Regulators Expression and Correlation Analysis

**Figure 2:**
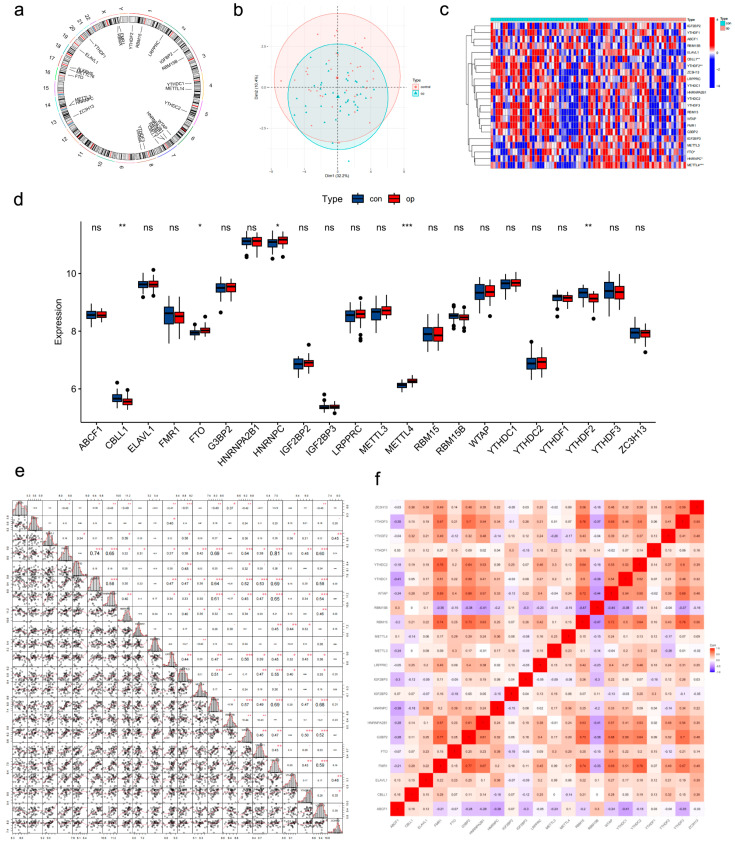
Identification of m6A regulators expression and correlation analysis. (**a**) Chromosomal locations of 22 m6A regulators. (**b**) PCA plot of osteoporosis and control samples. (**c**,**d**) Differential expression of m6A regulators between high- and low-BMD samples. (**e**,**f**) Correlation analysis among 22 m6A regulators in osteoporosis samples. *, *p* < 0.05; **, *p* < 0.01; ***, *p* < 0.001. ns, no significance.

First, we listed the 22 m6A regulators extracted from the previous literature in this study: *IGF2BP2*, *YTHDF1*, *ABCF1*, *RBM15B*, *ELAVL1*, *CBLL1*, *YTHDF2*, *ZC3H13*, *LRPPRC*, *YTHDC1*, *HNRNPA2B1*, *YTHDC2*, *YTHDF3*, *RBM15*, *WTAP*, *FMR1*, *G3BP2*, *IGF2BP3*, *METTL3*, *FTO*, *HNRNPC* and *METTL4*. Their chromosomal locations are shown (Figure 2a). We first analyzed the training dataset GSE56815, which consists of peripheral blood monocytes (PBMCs) from 40 high*-*bone mineral density (BMD) and 40 low-BMD individuals. Throughout this study, “high BMD” refers to control samples, and “low BMD” refers to osteoporosis samples. The PCA plot of osteoporosis and control samples is displayed (Figure 2b). We subsequently performed differential gene expression analysis on the training set. We found that five m6A regulators exhibited a great distinction between high- and low-BMD samples. Expression analysis showed that *FTO*, *HNRNPC*, and *METTL4* levels went up significantly while *CBLL1* and *YTHDF2* levels were downregulated in low-BMD samples compared to controls (Figure 2c,d). As we already knew that m6A regulators generally work as a network, we looked at how the 22 regulators in the low-BMD samples are connected to each other. The result revealed strong correlation between them (Figure 2e,f). For instance, the correlations between *FMR1* and *G3BP2* (r = 0.74), *FMR1* and *RBM15* (r = 0.69), *FMR1* and *YTHDC2* (r = 0.81), *G3BP2* and *YTHDC2* (r = 0.65), *RBM15* and *YTHDC2* (r = 0.70), and *RBM15* and *YTHDF3* (r = 0.68) are ranked top six.

### 2.2. Assessment of Immune Infiltration and Correlation Analysis

**Figure 3:**
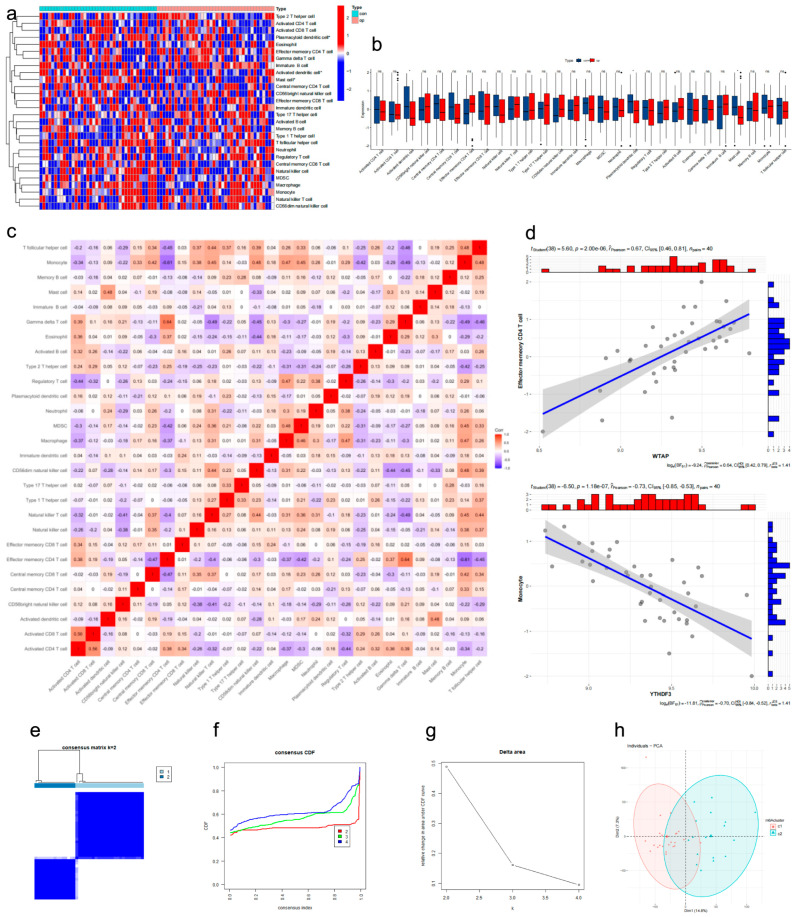
Evaluation of immune infiltration and correlation analysis. (**a**,**b**) Enrichment of immune cell types in high- and low-BMD samples. (**c**) Correlation network of immune cells. (**d**) Correlation plot. (**e**–**g**) Consensus clustering results (C1 = 23, C2 = 17, *p* < 0.001). (**h**) PCA plot. *, *p* < 0.05. ns, no significance.

To investigate the role of immune infiltration in osteoporosis, we evaluated the abundance of various immune cells in high- and low-BMD samples using the single-sample gene set enrichment analysis (ssGSEA) algorithm. Our analysis revealed significantly decreased enrichment of activated dendritic cells, plasmacytoid dendritic cells, and mast cells in the osteoporosis group compared to controls (Figure 3a,b). The correlation network among different immune cell types is shown in Figure 3c, which revealed a predominance of positive interactions. The correlations between m6A regulators and immune cells are displayed in the heatmap (Figure 3d).

### 2.3. Identification of Two m6A Consensus Clusters and Their Immune Features

**Figure 4:**
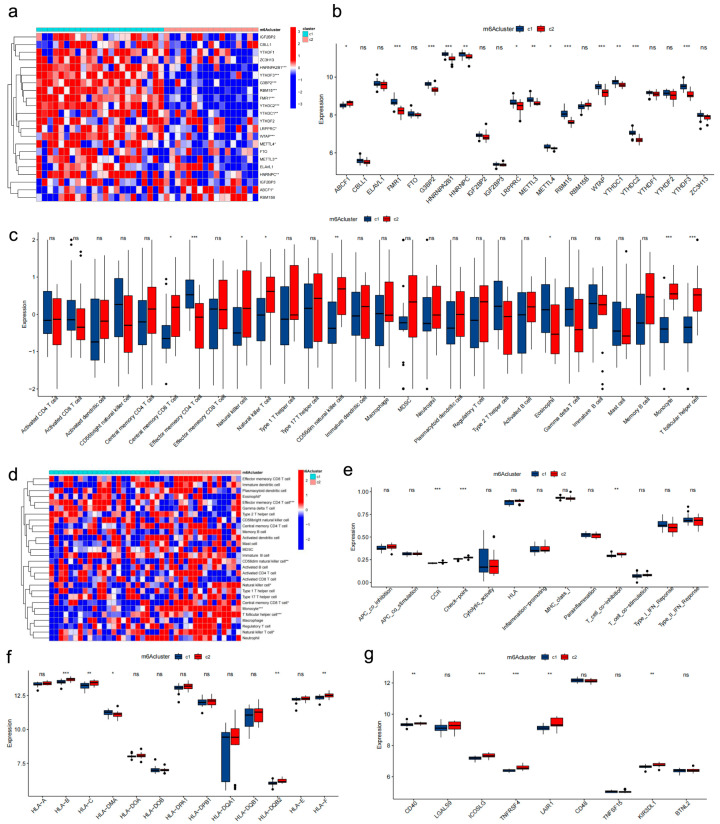
Construction of consensus clusters and IME analysis. (**a**,**b**) Expression heatmap of m6A regulators and immune cells between clusters. (**c**,**d**) Boxplot of immune cell enrichment in two clusters. (**e**–**g**) Immune pathways and HLA family gene expression between clusters. *, *p* < 0.05; **, *p* < 0.01; ***, *p* < 0.001. ns, no significance.

We used consensus clustering based on the 22 m6A regulators on the GSE56815 transcriptomic profiles; we identified two molecular subgroups (C1, *n* = 21; C2, *n* = 19) (Figure 3e–g). The principal component analysis (PCA) results (Figure 3h) showed that these two subgroups were clearly separate from each other. The large portion of m6A regulators (*LRPPRC*, *ABCF1*, *FMR1*, *G3BP2*, *HNRNPA2B1*, *HNRNPC*, *METTL3*, *METTL4*, *WTAP*, *YTHDC1*, *YTHDC2*, *YTHDF3*, *ZC3H13*, *RBM15*) was distinctly different in two m6A clusters (Figure 4a,b). Interestingly, *HNRNPC* and *METTL4* were significantly ectopically expressed in molecular clusters and different BMD samples. This suggests that *HNRNPC* and *METTL4* may occupy crucial positions at the center of the m6A regulatory network. More and more evidence demonstrated that IME play a crucial role in the progression of diseases. An assessment of 28 immune cell types, 13 immune pathways, and 13 HLA genes revealed significant immune activation in cluster C2. This was marked by significantly higher enrichment of central memory CD8 T cells, natural killer cells, natural killer T cells, CD56dim natural killer cells, monocytes, and T follicular helper cells, whereas cluster C1 was distinctly enriched with effector memory CD4 T cells and eosinophils (Figure 4c,d). Likewise, the majority of immune pathways, checkpoints, and HLA genes were upregulated in C2 (Figure 4e–g).

### 2.4. m6Ascore Subgroups and Associated Immune Landscape

**Figure 5:**
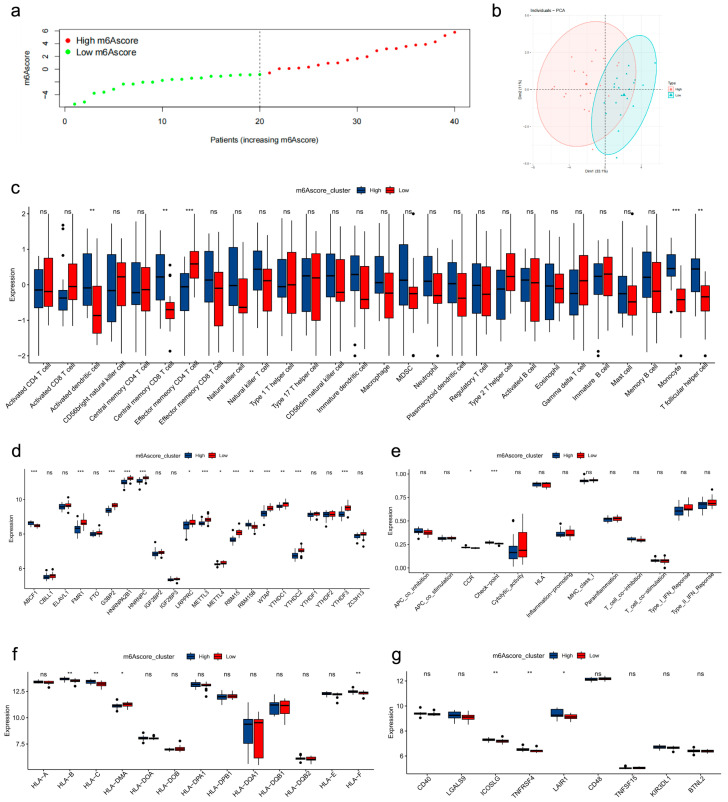
Construction of m6Ascore cluster and IME analysis. (**a**,**b**) Distribution of m6A score and PCA of score clusters (C1 = 20, C2 = 20, *p* < 0.001). (**c**) Immune cell enrichment between score clusters. (**d**) Expression of m6A regulators in high- and low-score clusters. (**e**) Immune pathway activity. (**f**,**g**) HLA gene expression in score clusters. *, *p* < 0.05; **, *p* < 0.01; ***, *p* < 0.001. ns, no significance.

First, 13 m6A regulators were screened out by differential expression analysis. The m6A score of each sample was calculated by PCA, then high- and low-score clusters were identified (Figure 5a,b). The immune landscape was also distinct: T follicular helper cells, monocytes, central memory CD8 T cells, and activated dendritic cells were highly enriched in these clusters, in stark contrast to the low-score cluster, which was uniquely characterized by higher enrichment of effector memory CD4 T cells (Figure 5c). Clusters with high m6A scores exhibited conspicuously low expression of key regulators, such as *FMR1*, *G3BP2*, *HNRNPA2B1*, *HNRNPC*, *LRPPRC*, *METTL3*, *METTL4*, *RBM15*, *RBM15B*, *WTAP*, *YTHDC1*, *YTHDC2*, and *YTHDF3* (Figure 5d). CCR and Checkpoint was enriched in high-score cluster (Figure 5e). In high m6A score clusters, genes, such as *ICOSLG*, *TNFRSF4*, *LAIR1*, *HLA−B*, *HLA−C*, and *HLA−F*, were all significantly upregulated; only *HLA−DMA* was downregulated (Figure 5f,g).

### 2.5. DEGs, Functional Enrichment, and PPI Network in Osteoporosis

**Figure 6:**
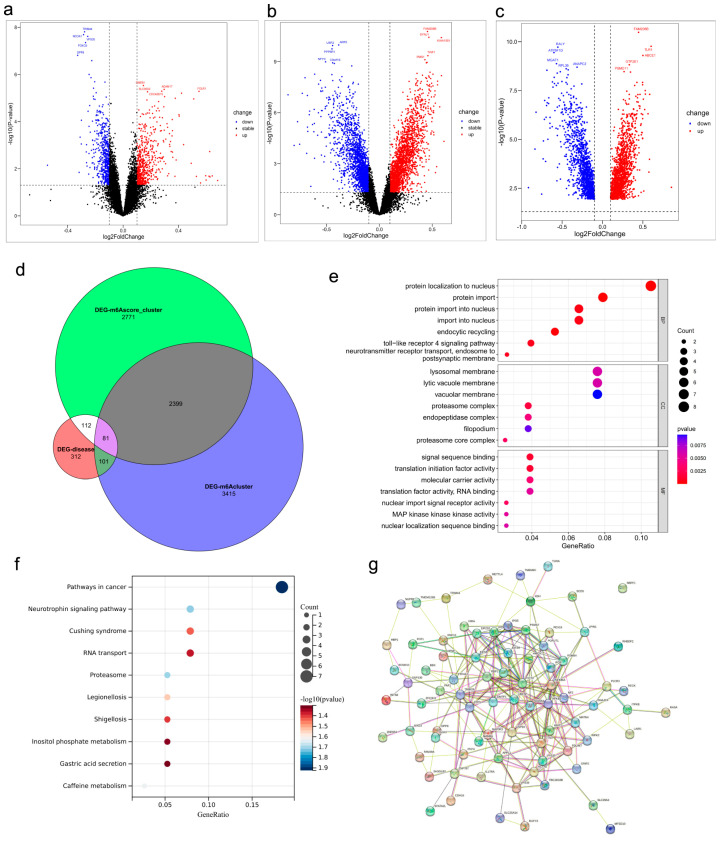
Differentially expressed genes and functional enrichment, PPI network. (**a**–**c**) Volcano plots of DEGs between high-/low-BMD, and score clusters. (**d**) Venn diagram of shared DEGs. (**e**) GO enrichment analysis of shared DEGs. (**f**) KEGG pathway enrichment. (**g**) PPI network of shared DEGs.

We screened out 312 DEGs between high- and low-BMD samples and 3415 DEGs between molecular clusters; Volcano plots were used to depict the details of these DEGs (Figure 6a–c). The intersection yielded 81 common DEGs (Figure 6d). These common DEGs were then subjected to GO and KEGG enrichment analysis to further understand the potential mechanisms involved in osteoporosis.

With regard to biological process, these genes were remarkably related to protein localization to nucleus, protein import. Cellular component suggested that 81 common DEGs were majorly located in lysosomal membrane and lytic vacuole membrane. In terms of molecular function, it indicated that signal sequence binding, and translation initiation factor activity were mainly enriched (Figure 6e). The result of KEGG pathway enrichment showed that these genes were mainly involved in cancer, neutrophil signaling pathway, Cushing syndrome, and RNA transport (Figure 6f). The PPI network of 81 genes is presented. It demonstrated that *DLD*, *LIAS*, *DBT*, *PDHB*, *DLAT* and *PDHA1* were the hub genes (Figure 6g).

### 2.6. Construction of Potential Biomarker Signature and Validation

**Figure 7:**
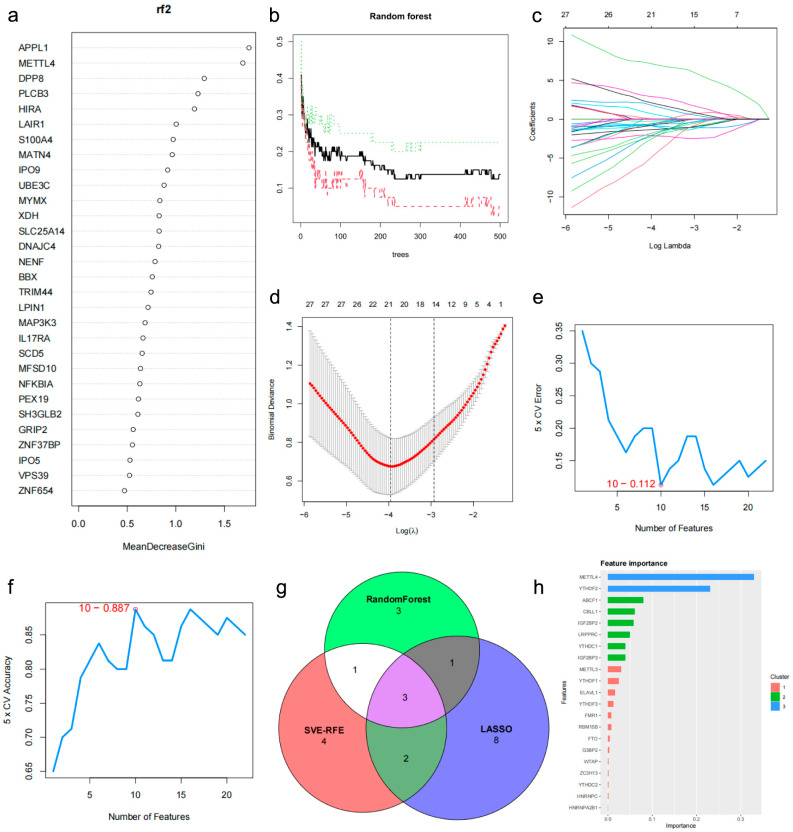
Construction of potential biomarker signature. (**a**,**b**) Random Forest model results. (**c**,**d**) LASSO regression results. (**e**,**f**) SVM-RFE algorithm results. (**g**) Overlap of candidate biomarkers from three algorithms. (**h**) XGboost importance ranking of m6A regulators.

First, we used Random Forest, LASSO regression, and SVM-RFE algorithms to identify biomarkers from 81 common DEGs for predicting osteoporosis. According to the Gini importance values, *APPL1*, *METTL4*, *DPP8*, *PLCB3*, *HIRA*, *LAIR1*, *S100A4*, and *MATN4* were ranked as the top eight by the Random Forest model (Figure 7a,b). Next, 14 key genes (*METTL4*, *HIRA*, *MATN4*, *IL17RA*, *XDH*, *PAF1*, *SLC29A3*, *NFKBIA*, *LAIR1*, *APPL1*, *IPO9*, *UBE3C*, *GRIP2*, *MAP3K3*) were screened as candidate genes by the LASSO algorithm (Figure 7c,d). Additionally, 10 genes (*METTL4*, *MAP3K3*, *IPO9*, *UBE3C*, *HIRA*, *GRIP2*, *GJA3*, *ZNF654*, *MATN4*, *EIF2S2*) were selected by SVM-RFE (Figure 7e,f). Finally, three genes (*METTL4*, *HIRA*, and *MATN4*) were selected as candidate biomarkers by overlapping the candidate genes from the three algorithms (Figure 7g).

XGBoost was chosen to identify the key m6A regulators with the greatest impact on osteoporosis. *METTL4* and *YTHDF2* were ranked at the top in terms of importance.

**Figure 8:**
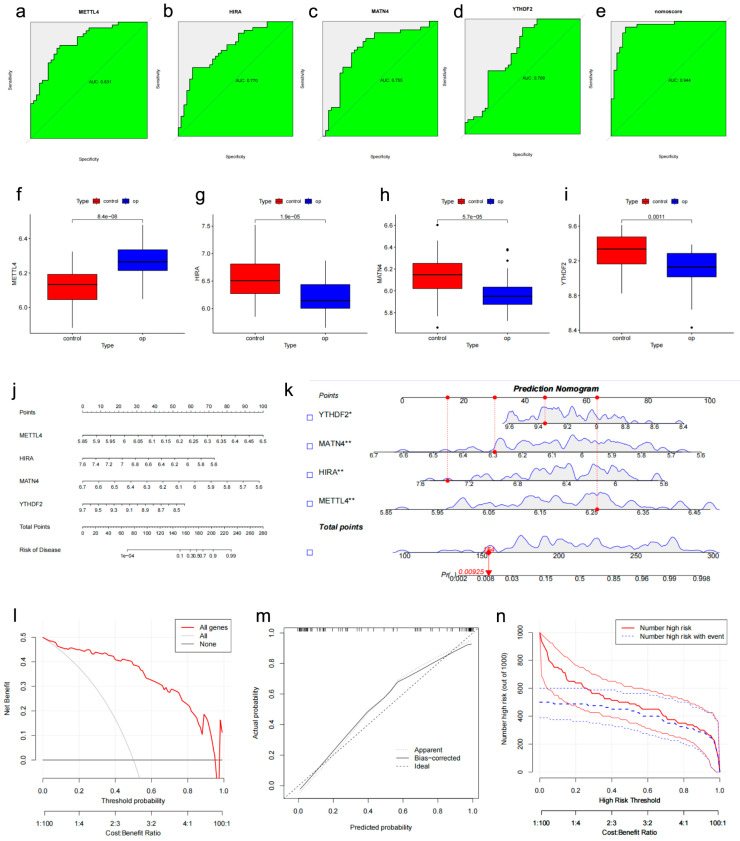
Potential biomarker signature construction and performance. (**a**–**d**) ROC curves of METTL4, YTHDF2, HIRA, and MATN4. (**e**) Nomogram ROC. (**f**–**i**) Expression of biomarkers. (**j**,**k**) Nomogram (**l**–**n**) DCA, calibration, and clinical impact curves. *, *p* < 0.05; **, *p* < 0.01.

**Figure 9:**
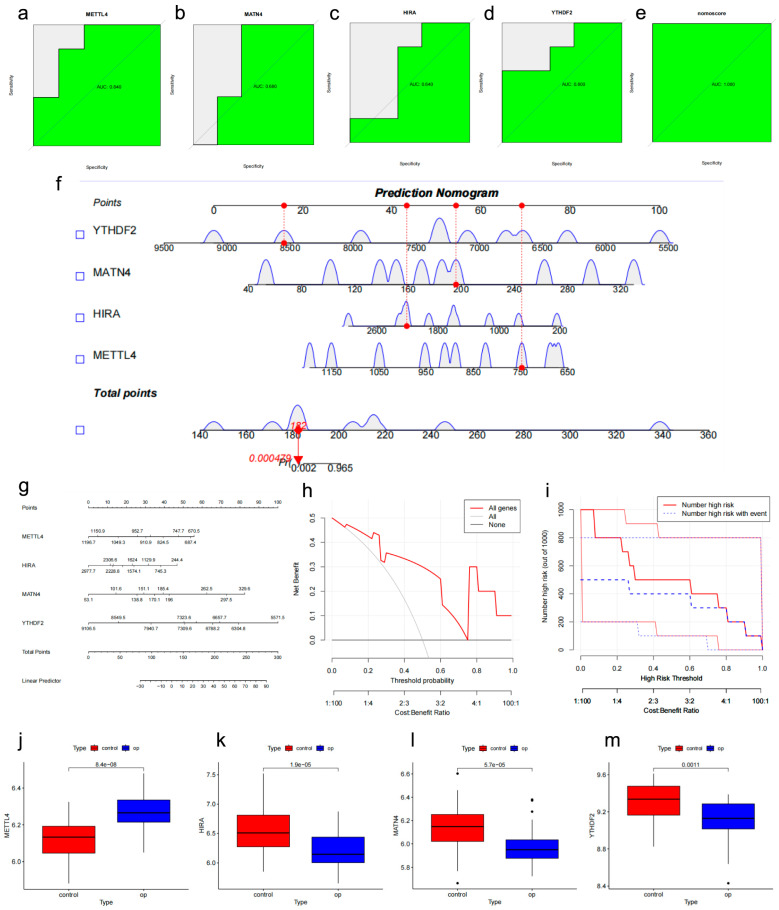
External validation of potential biomarker signature. (**a**–**d**) ROC curves in GSE35956. (**e**) Nomogram ROC. (**f**,**g**) Nomogram. (**h**,**i**) DCA and clinical impact curves. (**j**–**m**) Biomarker expression in GSE56814.

**Figure 10:**
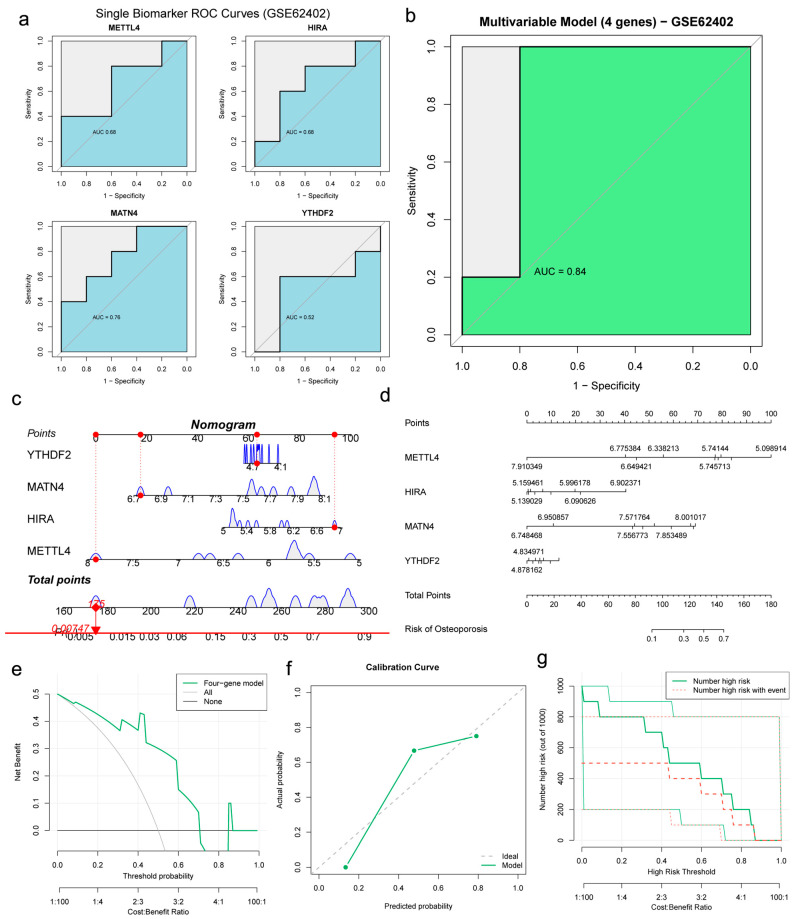
External validation of potential biomarker signature. (**a**,**b**) ROC curves in GSE62402. (**c**,**d**) Nomogram ROC. (**e**–**g**) DCA, calibration, and clinical impact curves.

Finally, the key m6A regulators (*METTL4*, *YTHDF2*) screened by the XGBoost algorithm (Figure 7h) and the candidate genes (*METTL4*, *HIRA*, and *MATN4*) selected from common DEGs were combined to construct a clinical nomogram. The AUC values for *METTL4*, *YTHDF2*, *HIRA*, and *MATN4* were 0.831, 0.709, 0.770, and 0.755, respectively (Figure 8a–d). The AUC value of the nomogram was favorable (Figure 8e). The differential expression of these candidate genes is shown (Figure 8f–i). The nomogram demonstrated greater predictive accuracy than any single biomarker (Figure 8j,k). DCA indicated that patients could benefit from the nomogram model (Figure 8l). Additionally, calibration curves and clinical impact curves confirmed the favorable performance of the potential biomarker signature in predicting disease occurrence (Figure 8m,n). Finally, we performed an exploratory assessment of the potential biomarker signature using the independent human mesenchymal stromal cells (hMSCs) dataset GSE35956. The AUC values for *METTL4*, *YTHDF2*, *HIRA*, and *MATN4* were 0.840, 0.800, 0.640, and 0.680, respectively (Figure 9a–d). The AUC value of the nomogram remained favorable (Figure 9e). The nomogram, DCA, and clinical impact curves all demonstrated the preliminary promise of the potential biomarker signature (Figure 9f–i). The differential expression of these candidate genes was validated in the GSE56814 dataset (Figure 9j–m). To further validate the potential biomarker signature in peripheral blood, we assessed its performance in the independent GSE62402 PBMCs dataset. The AUC values for *METTL4*, *YTHDF2*, *HIRA*, and *MATN4* were 0.68, 0.52, 0.68, and 0.76, respectively (Figure 10a). While *YTHDF2* showed limited individual predictive value in this dataset, the combined nomogram achieved an AUC of 0.84 (Figure 10b–d), with DCA, calibration, and clinical impact curves confirming its clinical utility (Figure 10e–g).

### 2.7. Pathway Enrichment Analysis of Candidate Biomarkers

**Figure 11:**
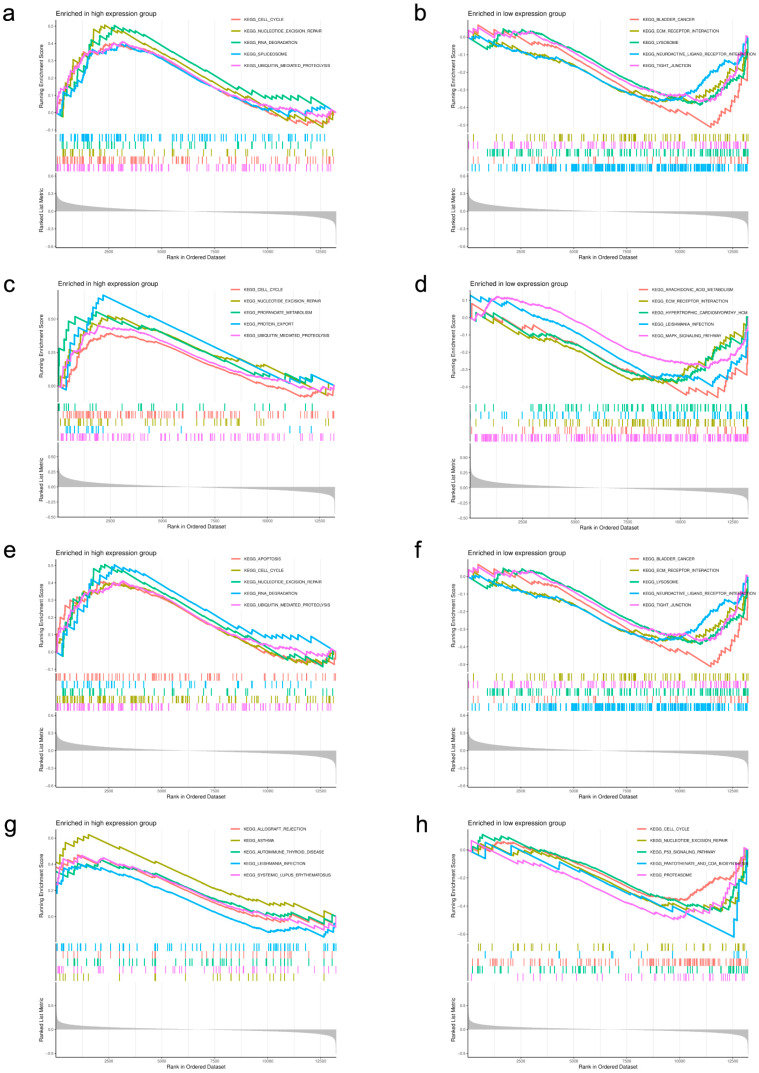
GSEA analysis. (**a**–**h**) GSEA for *METTL4*, *YTHDF2*, *HIRA*, and *MATN4*.

We performed analysis of GSEA in low-BMD samples to study the crucial molecular mechanism of potential biomarkers. The results of the GSEA were illustrated (Figure 11a–h).

The results showed that high expression of *HIRA* was enriched in apoptosis and cell cycle pathways, while low expression was linked to ECM–receptor interaction and lysosome. *MATN4* upregulation was associated with allograft rejection and asthma, and downregulation was related to cell cycle and p53 signaling. *METTL4* high expression was involved in cell cycle and spliceosome, and low expression was correlated with ECM–receptor interaction and tight junction. For *YTHDF2*, high expression was enriched in cell cycle and ubiquitin-mediated proteolysis, and low expression was involved in ECM–receptor interaction and MAPK signaling pathway.

**Figure 12:**
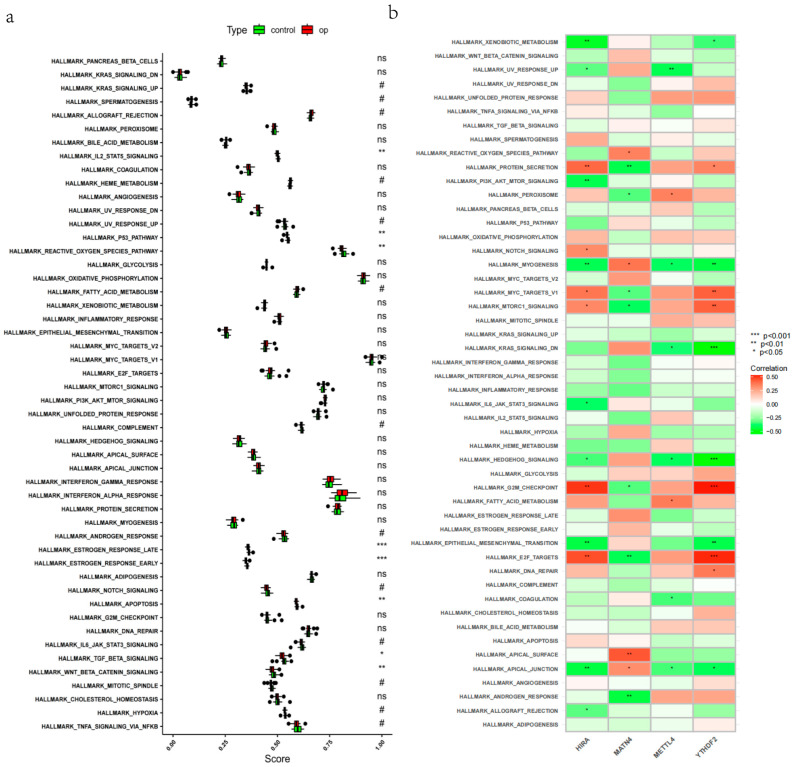
Pathway correlation. (**a**) GSVA pathway activity. (**b**) Biomarker–pathway correlation. *, *p* < 0.05; **, *p* < 0.01; ***, *p* < 0.001; ns, no significance. #, 0.05 ≤ *p* < 0.10.

The result of GSVA indicated that the difference between high- and low-BMD conditions was majorly involved in eight pathways (Figure 12a): IL2-STAT5 signaling, UV response up, p53 pathway, estrogen response late, estrogen response early, apoptosis, IL6-JAK-STAT3 signaling, and TGF-beta signaling. All these pathways were enriched in osteoporosis condition. Interestingly, several popular osteoporosis-related pathways were identified, such as UV response up, estrogen response early/late, and apoptosis.

The correlation between potential biomarkers and the above GSVA results is detailed in the figure (Figure 12b). Notably, *HIRA* was markedly correlated with Hallmark IL6-JAK-STAT3 signaling, while Hallmark reactive oxygen species pathway not only differed between control/osteoporosis but also positively correlated with biomarker *MATN4*.

### 2.8. Single-Cell Transcriptomic Profiling Reveals Cell-Type Specific Expression of Potential Biomarkers and Altered Intercellular Communication

**Figure 13:**
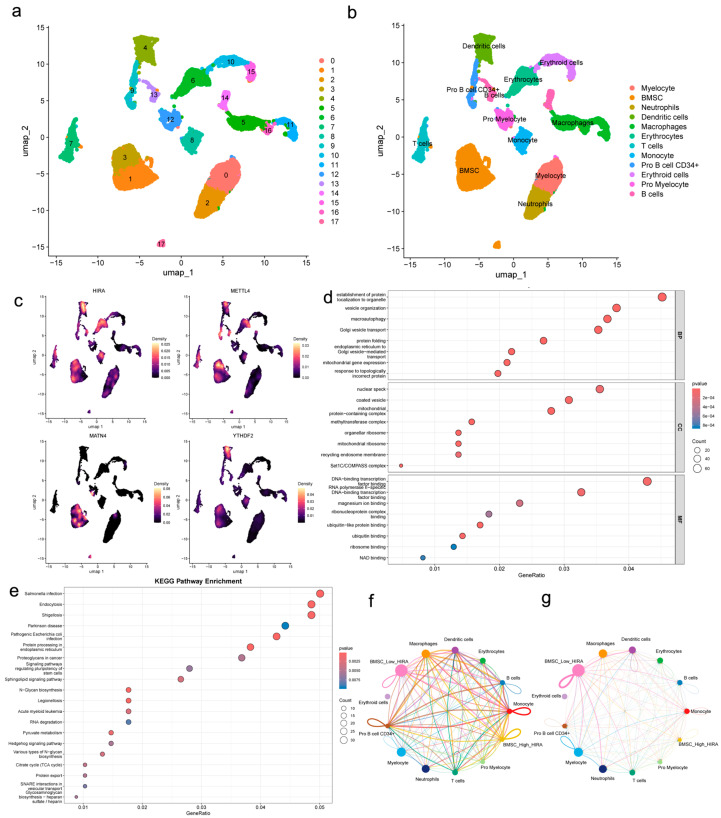
Single-cell transcriptomic analysis. (**a**) Unsupervised clustering. (**b**) Cell type annotation. (**c**) Expression distribution of *METTL4*, *HIRA*, *MATN4*, and *YTHDF2*. (**d**) GO enrichment. (**e**) KEGG pathway enrichment. (**f**) Cell–cell interaction count. (**g**) Cell–cell interaction strength.

To define the cellular origin of the four potential biomarkers and to explore their functional context, we analyzed the single-cell RNA-seq dataset GSE147287. After quality control and unsupervised clustering, 10 major cell populations were identified and visualized by UMAP (Figure 13a). Based on canonical markers and SingleR annotation, these clusters were assigned to known bone marrow cell types, including BMSCs, dendritic cells (DCs), T cells, monocytes, and others (Figure 13b).

Nebulosa density plots showed that *METTL4* and *MATN4* were predominantly enriched in the BMSCs cluster, whereas *YTHDF2* was almost exclusively expressed in dendritic cells. Notably, *HIRA* exhibited a dual distribution, with high expression detected in both BMSCs and dendritic cells (Figure 13c).

To further investigate the functional implications of *HIRA* expression heterogeneity, we subdivided BMSCs into *HIRA*-high and *HIRA*-low subgroups based on expression. Differential expression analysis between these subgroups identified 1583 significant genes. GO enrichment of these genes highlighted terms related to vesicle-mediated transport, mitochondrial protein complexes, and protein folding (Figure 13d). KEGG pathway analysis revealed enrichment in endocytosis, protein processing in endoplasmic reticulum, and several infection-related pathways (Figure 13e).

Cell–cell communication analysis using CellChat revealed 468 significant interactions involving 19 signaling pathways (Figure 13f). Comparison between *HIRA*-high and *HIRA*-low BMSCs subgroups showed distinct communication patterns (Figure 13g).

### 2.9. Experimental Validation of Potential Biomarkers in Independent Clinical Cohorts

**Figure 14:**
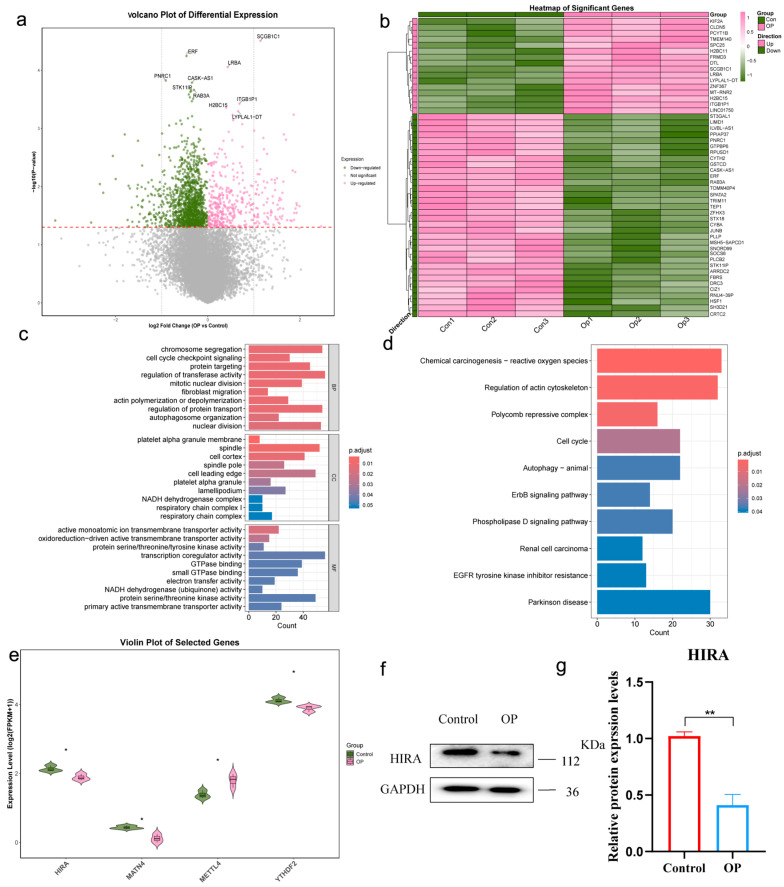
Experimental validation of potential biomarkers. (**a**) Volcano plot of DEGs from PBMCs transcriptome sequencing. (**b**) Heatmap of top DEGs. (**c**) GO enrichment analysis. (**d**) KEGG pathway enrichment analysis. (**e**) Violin plot of potential biomarkers. (**f**) Representative Western blot images of HIRA in bone tissue. (**g**) Quantitative analysis of HIRA protein expression. *, *p* < 0.05; **, *p* < 0.01.

To experimentally validate the potential biomarkers identified by bioinformatic analysis, we performed transcriptome sequencing on PBMCs from an independent cohort. Differential expression analysis identified significantly differentially expressed genes (Figure 14a). The heatmap of top DEGs demonstrated distinct transcriptional profiles that effectively segregated OP samples from controls (Figure 14b). GO and KEGG enrichment analyses of the DEGs were performed to explore the functional implications of the transcriptional changes (Figure 14c,d). Notably, the expression levels of the four biomarkers were all consistent with the expected directions in the OP group compared to controls (Figure 14e).

Western blot analysis of an independent bone tissue cohort confirmed that HIRA protein expression was decreased in the osteoporosis group (Figure 14f,g). This convergence of transcriptomic evidence from PBMCs and protein-level validation from bone tissue provides preliminary multi-level evidence of HIRA as a downregulated potential biomarker in osteoporosis.

## 3. Discussion

Osteoporosis pathogenesis fundamentally stems from disrupted bone metabolic equilibrium [21]. This vital balance relies on coordinated actions between bone-resorbing osteoclasts and bone-forming osteoblasts [22,23]. Osteoclasts develop as multinuclear entities within bone marrow to degrade osseous tissue. Conversely, osteoblasts differentiate from mesenchymal progenitors inhabiting periosteal and marrow stromal compartments, executing bone matrix synthesis and mineralization processes. The co-regulation of osteoclasts and osteoblasts requires precise regulation of mRNA, a process that is modulated by various epigenetic mechanisms, including N6-methyladenosine (m6A) [24]. m6A modifications mediate *ALP*, *Runx2*, and *VEGF* to regulate the proliferation, differentiation, and apoptosis of BMSCs, osteoblasts, and osteoclasts. Upregulation of *METTL3* promoted osteogenic differentiation and remedied dysfunction of bone mesenchymal stem cells by maintaining the stability of *RUNX2* [25,26].

This study systematically elucidated the fundamental role of m6A regulators in OP and their interaction with the IME. We observed significant upregulation of *FTO*, *HNRNPC*, and *METTL4*, alongside downregulation of *CBLL1* and *YTHDF2* in OP, indicating that the overall activation of m6A modification may facilitate the progression of OP. These regulators exhibited a significant correlation with the enrichment of immune cells, including effector memory CD4+ T cells and monocytes, thereby forming a potential immune epigenetic network that influences bone metabolism. Using consensus clustering, we found OP molecular subtypes (C1 and C2) with a lot of IME heterogeneity, which gave us a solid foundation for accurately classifying OP. We built a potential biomarker signature using *METTL4*, *HIRA*, *MATN4*, and *YTHDF2* by combining machine learning algorithms. This signature demonstrated favorable predictive performance in both internal and external datasets, showing that it could be used in clinical settings. Functional analysis showed that these markers were enriched in important pathways for bone metabolism, like IL-17 signaling, circadian rhythm, and apoptosis. This is a first step in figuring out how they work at the molecular level.

Recent studies have found that m6A methylation extensively regulates osteoblastic differentiation and bone remodeling [27]. For instance, the methyltransferase *METTL3* has been demonstrated to promote osteoblast formation and inhibit osteoclast differentiation by enhancing *FZD3* expression through m6A modification, thereby activating the *RUNX1* signaling axis [28]. Conversely, the piR48444/*METTL7A* axis negatively regulates osteogenesis by suppressing *BMP2* mRNA m6A methylation and its subsequent translation via the eIF4E complex, highlighting the delicate balance of m6A modifications in bone homeostasis [29]. Furthermore, m6A methylation has been shown to regulate bone remodeling through key signaling pathways including PTH/Pth1r, PI3K-Akt, and Wnt/β-catenin [30]. Tgether, these recent reports support our findings that m6A regulators are critically involved in osteoporosis pathogenesis, not only as potential risk biomarkers but also through complex post-transcriptional networks that govern the fate of bone cells.

Chen et al. reported that *METTL4* is an internal m6A methyltransferase (a writer) that mediates the regulation of RNA splicing [31]. A study by Min Shen and his colleagues indicated that upregulation of *METTL4* mediated ferroptosis through autophagy signaling pathway in hepatic stellate cells [32]. *METTL4* was also proven to regulate adipocyte differentiation by INSR signaling pathway, and affect lipid production and glucose up-taking [33]. Van den Homberg DAL found that *METTL4* was predominantly responsible for the increase in m6A modification in fibroblasts [34]. In our training dataset, *METTL4* played the key role in 22 m6A regulators. We found that *METTL4* was significantly upregulated in the osteoporosis group, with the lowest p value (*p* < 0.001) compared to control samples. In molecular clusters based on 22 m6A regulators, *METTL4* was upregulated in C1.

*YTHDF2* is the first reader protein of m6A regulators. It selectively recognizes m6A-containing mRNA and mediates the degradation of substrate RNA, suggesting a core role in mRNA degradation [35]. But, Deobrat Dixit et al. reported that *YTHDF2* was also capable of stabilizing specific types of transcripts in GSCs by certain unknown mechanisms [36]. In our study, the expression level of *YTHDF2* was downregulated in osteoporosis compared with high-BMD samples. And there was no difference in *YTHDF2* expression between molecular clusters and m6Ascore clusters. According to XGboost algorithms, *YTHDF2* was identified as key genes of m6A regulators involved in progression of osteoporosis. The AUC value of *YTHDF2* as a potential biomarker was 0.709 in the training dataset, and elevated to 0.800 in the external dataset. The performance of *YTHDF2* in potential biomarker signature also supported the center position in m6A regulators involved in osteoporosis.

A study found that *HIRA* regulates myogenic cell differentiation by depositing the histone variant H3.3 [37]. *HIRA* was also critical for maintaining the chromatin landscape of muscle stem cells and restricting the expression of myogenic genes [38]. Importantly, in cells deficient in fumarate hydratase 1, loss of *HIRA* led to elevated nucleotide metabolism, which promoted the activation of MYC and its target genes [39].

Ezura et al. reported that *MATN4*, an extracellular matrix protein, was significantly upregulated during chondrogenic differentiation of human synovium-derived MSCs. However, its promoter exhibited stable hypermethylation due to low CpG density, suggesting potential post-transcriptional regulatory mechanisms [40]. In our study, *MATN4* was identified as a downregulated potential biomarker in osteoporosis through integrated algorithms. GSEA analysis further linked *MATN4* downregulation to disruptions in circadian rhythm and nucleocytoplasmic transport pathways, which are critical for bone remodeling gene oscillations.

Consistent with our bioinformatic findings, *FTO* and *METTL4* were upregulated while *CBLL1* and *YTHDF2* were downregulated in OP. Strong correlations among *FMR1*, *G3BP2*, *RBM15*, and *YTHDC2* (r > 0.65) further suggested their central role in the m6A regulatory network under osteoporotic conditions. Overall, our findings underscore the significant role of m6A RNA methylation in the pathogenesis of osteoporosis, particularly through its interplay with the immune microenvironment. The differential expression of key regulators such as *METTL4* and *YTHDF2* highlights their potential importance in bone metabolism. Furthermore, the strong correlations observed between specific m6A regulators and immune cell populations suggest a coordinated mechanism influencing bone density.

The potential biomarker signature developed in this study, which integrates m6A regulators and immune-related DEGs, demonstrates promising but preliminary performance. Its successful validation in an external dataset supports the clinical applicability of these biomarkers for osteoporosis risk assessment.

Single-cell RNA sequencing added critical cellular resolution: *METTL4* and *MATN4* were enriched in BMSCs, *YTHDF2* in dendritic cells, while *HIRA* exhibited dual distribution in both. Notably, *HIRA* heterogeneity in BMSCs was associated with distinct transcriptional programs and cell–cell communication patterns, positioning it as a potential molecular bridge between osteogenic dysfunction and immune dysregulation. Integrating the single-cell resolution with our bulk-derived risk signature reveals that the candidate biomarkers are not randomly distributed but are enriched in biologically relevant cell types. This convergence strengthens the biological plausibility of the signature. Notably, the heterogeneity of *HIRA* expression in BMSCs was associated with distinct cell–cell communication patterns, suggesting that *HIRA* may serve as a molecular bridge between osteogenic dysfunction and immune dysregulation in osteoporosis.

In the experimental validation section, we adopted a hierarchical validation strategy to assess the translational relevance of our findings. Transcriptome sequencing was performed on PBMCs to evaluate the potential of the signature as a minimally invasive liquid biopsy, capturing the systemic epigenetic–immune status from circulating cells. Second, to validate the signature in osteogenic progenitors, we analyzed an external hMSC dataset; hMSCs are the primary bone-forming cells whose dysfunction directly drives osteoporosis pathogenesis. Third, Western blot analysis was conducted on bone tissue from independent clinical cohorts to confirm the presence of candidate biomarkers at the actual disease-affected site, providing direct evidence in the target organ. The transcriptomic data confirmed significant downregulation of *HIRA* mRNA in osteoporosis patients, and this finding was further corroborated at the protein level in bone tissue.

Limitations of this study should be acknowledged. First, our analyses were primarily based on public datasets and in silico predictions, which, although complemented by independent experimental validation, require further functional interrogation. The causal relationships between the identified m6A regulators, immune cell alterations, and bone loss remain to be established through rigorous in vitro and in vivo loss-/gain-of-function studies. Second, the study involved heterogeneous sample types across analytical stages—including peripheral blood monocytes, mesenchymal stem cells, peripheral blood mononuclear cells, and bone tissue—which may limit direct cross-comparability. Most importantly, all experimental validation cohorts were critically small (*n* ≤ 6 per group), which severely limits statistical power and generalizability. Our findings are therefore preliminary and should be interpreted with caution. Large-scale, prospective, multi-center studies using consistent cell types are urgently needed before any clinical application can be considered. Third, the training datasets used in this study (GSE56815) predominantly consist of female samples; therefore, the applicability of the identified risk signature to male osteoporosis remains to be validated in future studies with male-specific or mixed-gender cohorts. Fourth, the immune cell enrichment analyses presented in this study were derived from transcriptomic data using computational algorithms and represent relative enrichment of gene signatures rather than direct quantification of cell infiltration. Finally, the precise molecular mechanisms by which *HIRA* and *METTL4* regulate osteoblast or osteoclast activity—and how they integrate with the m6A modification network—merit dedicated mechanistic exploration. Future studies should also investigate the therapeutic potential of targeting these regulators in preclinical osteoporosis models.

## 4. Materials and Methods

### 4.1. Bioinformatics Analyses and Experimental Validation

#### 4.1.1. Data Gathering and Preprocessing

We sourced the transcriptomic data from the NCBI Gene Expression Omnibus (GEO) (https://www.ncbi.nlm.nih.gov/geo/query/acc.cgi, accessed on 25 December 2026), utilizing the GEOquery package for data acquisition and curation. The mRNA expression profiles and clinical features of GSE56815 (GPL96 platform), GSE35956 (GPL570 platform), and GSE56814 (GPL5175 platform) were downloaded using the “GEOquery” package. We transformed all platform probe information into corresponding gene symbols. The GSE56814 dataset comprised 42 high-BMD and 31 low-BMD monocyte samples. Dataset of GSE56815 was composed by 40 high- and 40 low-BMD monocyte samples, and set as training dataset. GSE35956 dataset contained 5 hMSCs samples from non-osteoporotic donors, and 5 hMSCs samples from osteoporotic donors, and set as external test dataset. Additionally, the GSE62402 dataset (GPL10526 platform) was included as an independent validation dataset. This dataset comprises PBMCs samples.

#### 4.1.2. Comparison of m6A Regulators and Immune Infiltration

We looked at 22 m6A regulators’ expression levels in the GSE56815 dataset to see how they were different between samples with high and low BMD. We used the ssGSEA method in the GSVA package to evaluate the enrichment of 28 immune cell types, with a significance level of *p* < 0.05. It was also possible to use either the Pearson or Spearman methods for correlation analysis to look at the links between m6A regulators and immune cells.

#### 4.1.3. Construction of Consensus Clusters and Immune Microenvironment (IME) Analysis

In order to describe the molecular diversity of osteoporosis, the 40 low-BMD samples were put into separate molecular groups by using consensus clustering to look at the expression of 22 m6A regulators. Principal Component Analysis (PCA) showed that these clusters were split up. In the next step, a detailed comparison was made to see how the m6A regulator expression and IME traits were different between the clusters. There were 13 immune pathways, 28 immune cells, and 13 HLA family genes in this group.

#### 4.1.4. Construction of m6Ascore_cluster and IME Analysis

First, we obtained m6A regulators differentially expressed in osteoporosis and control, and PCA helped us figure out what each sample’s m6A value was. The samples were then split into two groups based on the middle score: high-m6Ascore and low-m6Ascore. Then, we carefully looked at how the expression of the 22 m6A regulators and the IME components (immune cells, immune pathways, and HLA family genes) were different in these two groups.

#### 4.1.5. Identification of Differentially Expressed Genes, Functional Enrichment, and PPI Network Construction

After removing batch effects, we identified DEGs between high- and low-BMD samples, as well as between the two molecular clusters and between the two score clusters, using the “limma” package. To screen DEGs, |Log_2_FC| had to be greater than 0.1 and adj.*p*.Val had to be less than 0.05. It was shown in the form of a volcano map. The “clusterProfiler” package was used to do functional enrichment analysis on the genes that were present in all three DEG sets. KEGG pathways were used in this study. GO terms are biological processes, cellular parts, and molecular functions. The “ggplot” package was used to illustrate the above analysis results.

In order to understand the inner communication of common DEGs, we used STRING database (http://string-db.org/, accessed on 25 December 2025) to identify protein interaction networks.

#### 4.1.6. Construction of Potential Biomarker Signature

We selected genes from common DEGs as candidate biomarker by Random Forest, and LASSO regression algorithm was also used to narrow down common DEGs with 10-fold cross validation, and lambda.1se. Subsequently, potential biomarkers were found by putting genes from two different methods together. With same criterion and strategies, Random Forest, and LASSO regression algorithm were used to selected candidate biomarkers from 22 m6A regulators.

Candidate biomarkers from common DEGs and 22 m6A regulators were combined together, and were employed to construct a nomogram for predicting the occurrence of osteoporosis. ROC curves with Area Under the Curve (AUC) value, DCA curves and clinical impact curves were drawn to validate the potential clinical value of potential biomarker signature. Finally, we validated the m6A nomogram in GSE35956 datasets. The nomogram was further validated in the independent GSE62402 PBMCs dataset using the same ROC, DCA, and calibration curve analyses.

Finally, boxplot was employed to illustrate the differential expressed level of candidate biomarkers in high- and low-BMD samples in GSE56815 datasets.

#### 4.1.7. GSEA of Biomarkers and GSVA of OP-Related Pathways

For further exploration of the potential mechanism of potential biomarkers involved in osteoporosis, we performed GSEA. Based on expression level of biomarkers, all osteoporosis samples were put into high and low groups, then DEGs were screened out. HALLMARK gene set was downloaded from was obtained from the Molecular Signatures Database (MSigDB), and GSEA of each biomarker was conducted; the first and last five pathways were illustrated by “gseaplot2” package.

Meanwhile, GSVA was conducted to evaluate the difference enrichment pathways between control and osteoporosis samples. A *t*-test was used to compare the activity of 50 key pathways between normal and osteoporosis samples by finding their enrichment scores. After that, a Pearson correlation analysis was performed to look into the links between the biomarkers and the pathways that had been greatly changed.

#### 4.1.8. Single-Cell RNA-Seq Data Processing

GSE147287 was processed with Seurat (v4.0). Cells with <200 genes or >15% mitochondrial reads were excluded. After normalization, the top 1500 variable genes were selected. Batch effects were corrected with Harmony. UMAP and clustering (resolution = 0.6) were performed using the first 20 PCs. Cell types were annotated with SingleR. Nebulosa was used for gene expression visualization. BMSCs were stratified by median HIRA expression. Differential expression (Wilcoxon, *p*.value, |log2FC| > 0.25), GO/KEGG enrichment (clusterProfiler, *p*.adjust < 0.2), and CellChat analyses were performed as described.

#### 4.1.9. Transcriptomic and Western Blotting Validation of Potential Biomarkers in Independent Clinical Cohorts

##### Collection of Peripheral Blood Mononuclear Cells and Bone Tissue Samples

For transcriptome sequencing validation: An independent cohort of 6 age- and sex-matched participants was enrolled, including 3 OP patients (BMD T-score ≤ −2.5) and 3 normal controls (BMD T-score ≥ −1.0). Peripheral venous blood (7 mL) was collected into EDTA anticoagulant tubes after overnight fasting. PBMCs were isolated within 2 h using human PBMC separation medium (Solarbio, China) by density gradient centrifugation, washed twice with D-Hanks‘ solution, and stored at −80 °C for RNA extraction.

For Western blot validation: An independent cohort of 6 patients undergoing spinal surgery for lumbar degeneration was enrolled. Discard trabecular bone fragments were collected from the posterior spinal elements. Based on preoperative BMD T-scores, patients were stratified into an OP group (*n* = 3) and a Con group (*n* = 3). Bone pieces were promptly cleared of soft tissue and marrow, rinsed with ice-cold PBS, snap-frozen in liquid nitrogen, and stored at −80 °C for protein extraction.

##### RNA Extraction, Library Construction, and Transcriptome Sequencing of PBMCs

Total RNA was extracted from PBMCs using TRIzol reagent (Magen, R4801-02). RNA concentration and purity were measured with a NanoDrop spectrophotometer (Thermo Fisher Scientific; Waltham, MA, USA), and RNA integrity was assessed using an Agilent 4150 system. Samples with RIN ≥ 7.0 were used for library preparation. Strand-specific RNA sequencing libraries were prepared from 1 μg of total RNA using the NEBNext^®^ Ultra™ II RNA Library Prep Kit for Illumina^®^ (NEB; MA, USA) according to the manufacturer‘s protocol. Briefly, mRNA was enriched with oligo(dT) beads, fragmented, and reverse-transcribed. The double-stranded cDNA was end-repaired, A-tailed, ligated with indexed adapters, size-selected, and PCR-amplified. Library quality was assessed on an Agilent 4150 system(Santa Clara, CA, USA), and equimolar pooled libraries were subjected to 150 bp paired-end sequencing on an Illumina NovaSeq 6000 platform(Illumina, San Diego, CA, USA).

##### Sequencing Data Analysis

Raw reads were processed with fastp for quality control and adapter trimming. Clean reads were aligned to the human reference genome (GRCh38) using HISAT2. Gene expression levels were quantified as FPKM using StringTie. Differential expression analysis between OP and Con groups was performed with DESeq2. GO and KEGG enrichment analyses were conducted using clusterProfiler (v4.0.2).

##### Western Blot Analysis

Bone tissue samples were ground in liquid nitrogen and lysed in RIPA buffer containing 1% SDS and protease inhibitor cocktail. After sonication and centrifugation (12,000× *g*, 10 min, 4 °C), protein concentration was determined by BCA assay. Equal amounts of protein (30 μg) were separated by 10% SDS-PAGE, transferred to PVDF membranes, blocked with 5% non-fat milk, and incubated overnight at 4 °C with primary antibodies against HIRA (1:1000, Abcam, ab129169) and GAPDH (1:5000, Proteintech, 60004-1-Ig). Membranes were then incubated with HRP-conjugated secondary antibodies (1:5000, ZSGB-BIO) for 1 h at room temperature. Protein bands were visualized using an ECL detection system (Tanon) and quantified with ImageJ software (version 1.54f). HIRA expression was normalized to GAPDH.

### 4.2. Statistical Methods

We employed R for bioinformatic analyses. Inter-group comparisons utilized the Wilcoxon rank-sum test, while Spearman’s rank correlation assessed associations. Statistical significance was defined as *p* < 0.05. All analyses were conducted using GraphPad Prism 9.0 (GraphPad Software) and R version 4.1.3 (https://www.r-project.org; accessed on 25 December 2025).

#### Declaration of Generative AI Use

Generative artificial intelligence (GenAI) was not used in any part of this study, including study design, data collection, analysis, interpretation, or the preparation of the manuscript.

## 5. Conclusions

This study enhances the comprehension of the epigenetic–immune cross-regulation mechanism in OP and introduces a promising potential biomarker signature, offering a novel perspective for the early risk stratification and targeted treatment research of OP.

## Data Availability

The datasets generated and/or analyzed during the current study are available from the corresponding author on reasonable request. Publicly available datasets (GSE56815, GSE35956, GSE56814, GSE147287, GSE62402) were downloaded from the GEO.

## Abbreviations

The following abbreviations are used in this manuscript:

m6A	N6-methyladenosine
OP	Osteoporosis
BMD	Bone Mineral Density
IME	Immune Microenvironment
DEG	Differentially Expressed Gene
GSEA	Gene Set Enrichment Analysis
GSVA	Gene Set Variation Analysis
PCA	Principal Component Analysis
AUC	Area Under the Curve
ROC	Receiver Operating Characteristic
BMSCs	Marrow Mesenchymal Stem Cells
hMSCs	human mesenchymal stromal cells
DCA	Decision Curve Analysis
PBMCs	Peripheral Blood Monocytes
